# Mini-FLOTAC, Kato-Katz and McMaster: three methods, one goal; highlights from north Argentina

**DOI:** 10.1186/1756-3305-7-271

**Published:** 2014-06-14

**Authors:** Beatrice Barda, Pamela Cajal, Eliana Villagran, Ruben Cimino, Marisa Juarez, Alejandro Krolewiecki, Laura Rinaldi, Giuseppe Cringoli, Roberto Burioni, Marco Albonico

**Affiliations:** 1 Laboratory of Microbiology San Raffaele Hospital, Milan, Italy; 2 Instituto de Investigaciones en Enfermedades Tropicales, Universidad Nacional de Salta – sede, Orán, Argentina; 3 Section of Veterinary Parasitology and Parasitic Diseases, University of Naples Federico II, Naples, Italy; 4 Ivo de Carneri Foundation, Milan, Italy

**Keywords:** Soil-transmitted helminths, Diagnostic techniques, Mini-FLOTAC technique, Kato-Katz thick smear, McMaster method

## Abstract

**Background:**

Copro-parasitological diagnosis is still a challenge in management of helminth infections at individual and community levels in resource-limited settings.

The aim of our study was to compare the performance of three quantitative techniques: Kato-Katz, McMaster and Mini-FLOTAC methids. The study was carried out in Oran, Northern Argentina.

**Methods:**

200 schoolchildren were enrolled to provide a single stool sample, which was tested for helminth infections with Kato-Katz, McMaster and Mini-FLOTAC methods. The Mini-FLOTAC was performed with two flotation solutions (FS2 saturated saline and FS7 zinc sulphate). Preparation and reading time for each of the three methods was calculated both when processing single and multiple samples.

**Results:**

Out of 193 schoolchildren examined, 40% were positive for any helminth infection by any method; the most prevalent was *Hymenolepis nana* (23%) followed by *Ascaris lumbricoides* (17%) and a third group of less prevalent helminths: *Enterobius vermicularis, Trichuris trichiura* and hookworms (11% all together). Mini-FLOTAC FS2 was more sensitive than FS7 for *H. nana* (93% vs 78%) and for other helminths (85% vs 80%), whereas FS7 was more sensitive for *A. lumbricoides* (87% vs 61%). Kato-Katz method was more sensitive than McMaster method for *A. lumbricoides* (84% vs 48%) and for other helminths (48% vs 43%) except for *H. nana* (49% vs 61%). As for egg counts, Mini-FLOTAC FS2 reported 904 eggs per gram of faeces (EPG) for *H. nana* (vs 457 with McMaster and 111 with Kato-Katz) and 1177 EPG for *A. lumbricoides* (vs 1315 with Kato-Katz and 995 with McMaster); FS2 detected the highest EPG for both *H.nana* and *A.lumbricoides* (904 vs 568 and 1177 vs 643 respectively), the differences were not statistically significant. The technique feasibility was calculated: Kato-Katz mean time was 48 minutes/sample, Mini-FLOTAC 13 minutes/sample and McMaster 7 minutes/sample. However, especially for Kato-Katz and Mini-FLOTAC, the mean time (min/sample) decreased significantly when processing multiple samples.

**Conclusions:**

Mini-FLOTAC is a promising technique for helminth diagnosis, it is more sensitive than Kato-Katz and McMaster for *H. nana* and as sensitive as Kato-Katz and more sensitive than McMaster for *A. lumbricoides* identification*.* Egg counts differences although relevant, did not reach statistical significance.

## Background

Soil-transmitted helminths (STH) (*Ascaris lumbricoides, Trichuris trichiura, Ancylostoma duodenale/Necator americanus*) infections still represent a great burden in public health and affect almost two billion people worldwide [1,2].

Mapping for STH prevalence has been identified as an important issue for decisions on preventive treatment in order to reach the goal of reducing the prevalence in high risk areas to less than 20% and to eliminate heavy intensity infections [1-3].

A recent review published by Pan American Health Organization focused on mapping prevalence of STH infections in areas of Latin America lacking epidemiological data, and summarized several surveys conducted in the last 20 years [4]. As expected, prevalence in Central and South America was fairly high; in Argentina, STH were detected especially in the regions of Salta and Cordoba with a prevalence of 20-67% in pre-school age children and 6-71% in school age children, the most frequent helminth being *A.lumbricoides*[4].

In 2007 Menghi *et al.*[5] conducted a survey in Tartagal, Salta Province, on STH and protozoa detecting a prevalence of 94.6% in the aboriginal community and the most common helminths were hookworms (58%), *Hymenolepis nana* (31%) and *Strongyloides stercoralis* (24%). These data were confirmed in a recent review by Socias *et al*. [6]. These results varied not only by geographical location, but also by the diagnostic methods used. One of the greatest limitations in parasite detection is the lack of a standard, sensitive and low-cost technique. This hinders different researchers from adopting the same method and comparing results. Efforts have been recently made in order to find a parasitological method that meets these features, and WHO is presently evaluating standard methods both for mapping and for monitoring efficacy of anthelminthic drugs and the impact of preventive chemotherapy in STH control programmes [7].

Kato-Katz is the quantitative technique recommended for the diagnosis of STH [8]. McMaster [9] and FLOTAC [10] techniques are used both in veterinary and human parasitology for faecal egg count of helminth parasites. The Mini-FLOTAC method [11] has been recently developed with the aim of combining sensitivity and low costs, in order to allow laboratories in resources-limited settings to rely on a good quantitative method both for diagnostic and epidemiological purposes. The aim of this study was to compare Kato-Katz and McMaster methods, with the newly developed Mini-FLOTAC technique for the diagnosis of intestinal helminth infections in a setting where resources are scarce.

In addition to comparing diagnostic accuracy, this study also focused on the affordability of this innovative technique and on its transferability to peripheral laboratories, in order to improve diagnosis and facilitate parasitological monitoring of STH control programmes.

## Methods

### Study site

San Ramon de la Nueva Oran is located in Northern Argentina, Salta Province, 32 km south from the Bolivian border. It is surrounded by rivers and therefore agriculture is the main economic resource for the population. The last National census in 2010 registered approximately 67,000 inhabitants. The climate is subtropical, characterized by a dry (April-October) and a humid (November- March) season. This survey was carried out in August-November 2012, during late winter and spring.

The Laboratory of Parasitology and Tropical Medicine is situated at the local branch of the National University of Salta; specimens from the primary care service are directed there for parasitological examinations free of charge.

### Study population and selection of samples

This study was carried out examining stool samples from one of the primary schools of the town. The school was randomly chosen among the schools in the suburbs of Oran where STH were thought to be more widespread. All children in the school were examined for STH infections to reach 200 children. This number was calculated, assuming a prevalence of STH of about 50% from previous studies, in order to have at least 100 positive children, to allow a power of 80% and a difference of 20% between the diagnostic methods with a 95% confidence level [11-13].

### Parasitological methods

All stool samples were collected and analyzed within 24 hours with the three different techniques: Kato-Katz, McMaster and Mini–FLOTAC.

The Kato-Katz was performed using the 41.7 mg template, according to WHO recommendations [8].

McMaster method was performed as the standard procedure: 2 g of faeces were filtered and homogenized with 30 ml of saturated saline. Two flotation chambers (1 ml each) were filled for each sample and three minutes were needed for the eggs to float. Eggs were counted and multiplied by 50 to have the eggs per gram of faeces (EPG) [9].

Mini-FLOTAC is a new diagnostic method based on flotation of the eggs. Two chambers (1 ml each) are placed in the base and surmounted by a reading disc. The Mini-FLOTAC is part of a kit together with the fill-FLOTAC, a plastic device used to homogenize, filter and pour the sample in the flotation chambers [11]. The floatation solutions (FS) used were the same described in the FLOTAC protocols [10]. For each sample two Mini-FLOTAC were performed, one with FS2 (saturated sodium chloride; density = 1.20) and one with FS7 (zinc sulphate; density = 1.35). Two grams of stool were weighed and diluted with 2 ml of 5% formalin, and thoroughly homogenized and filtered. The suspension (2 gram of stool + 2 ml of formalin) was directly added to 36 ml of the FS2 and to 46 ml of FS7. Ten minutes were needed for the eggs to float before translating the reading disc. Eggs of intestinal helminths were detected and counted within the grid (sensitivity of 10 eggs/gram for FS2 and 12.5 eggs/gram for FS7).

Intensity of infection for *A. lumbricoides* was considered according to WHO guidelines: light infection <5000 EPG, moderate 5000–49 999 EPG and heavy ≥50 000 EPG [12].

The feasibility of the 3 techniques was evaluated on a total number of 100 samples randomly assigned to 4 experienced laboratory technicians. The time needed to prepare the samples and to clean the devices was measured six times. The preparation period started when the samples were weighed and ended when the sample was ready for examination. During the cleaning period all used materials were either disposed, for single use components, or cleaned in the case of recyclable devices. Examination of the slides or chambers was timed individually for all samples. Each sample was examined with all techniques by the same laboratory technician.

### Statistical analysis

Data were analyzed by EPIDAT version 3.1 (Software for Epidemiologic Analysis of Tabulated Data, Xunta de Galicia and Pan American Health Organization, PAHO/WHO). Prevalence, sensitivity (Se), negative predictive value (NPV) and Kappa index (KI) for each method (Kato Katz, McMaster, Mini-FLOTAC FS2 and Mini-FLOTAC FS7) were calculated. Prevalence was defined as proportion of positive samples over total samples analyzed. A positive sample was defined if positive with any parasitological method, while a negative sample was considered negative if negative with all methods; this criterion was defined our “gold standard”. Sensitivity (i.e., proportion of true-positives among those infected) and NPV (proportion of negative results among un-infected subjects) were calculated using 2×2 contingency tables in relation to our “gold standard”. The agreement between the results of each method was calculated with the KI. Interpretation of KI was as follows: ≤0 = poor, 0.01-0.20 = slight, 0.21-0.40 = fair, 0.41-0.60 = moderate, 0.61-0.80 substantial, and 0.81-1.00 almost perfect agreement [14].

### Ethical considerations

The study was reviewed and approved by the Ethics Committee of the Faculty of Medicine, San Raffaele Hospital, Milan, Italy. A separate ethical clearance was obtained from the San Ramon de la Nueva Oran Hospital Management Board. The medical staff met school authorities and parents/guardians before enrolling their children in the survey and verbal informed consent was sought. After the survey, all schoolchildren were treated with mebendazole 500 mg in single dose according to the WHO recommendations for preventive chemotherapy [3]. Data were kept anonymous and patients were identified by code; the study data were safely filed and stored in a cabinet within the data management unit of the research site and remained confidential.

## Results

### Parasitological results

Out of two hundred children who were enrolled in the survey, 193 provided the stool samples. The enrolled children aged between 5 and 13 years old (mean 10 years), and 82 (42.5%) were males; 78 children (40%) were found positive for at least one helminth infection. Forty five (23%) children were found positive for *Hymenolepis nana,* 32 (16.6%) were positive for *Ascaris lumbricoides,* few others were found positive for other helminths: *Enterobius vermicularis, Trichuris trichiura* and hookworms (6%, 3% and 1.5%, respectively). For *A. lumbricoides* only two heavy infections were detected, nine were moderate and twenty-one light.

### Technique sensitivity

**Figure 1:**
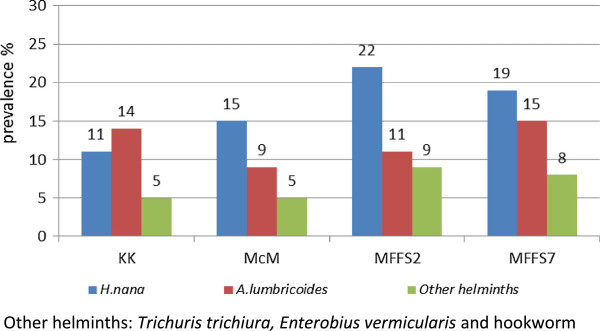
Prevalence of helminth infections with Kato-Katz (KK), McMaster (McM), Mini-FLOTAC FS2 (MFS2) and Mini-FLOTAC FS7 (MFFS7) methods.

Each sample was analysed with the three different techniques and results are shown in Figure 1 and Table 1; the most sensitive technique for *H.nana* was Mini-FLOTAC FS2 (92.7%) followed by FS7 (77.8%), McMaster (60.5%) and Kato-Katz (49.0%); for *A.lumbricoides* the most sensitive was Mini-FLOTAC FS7 (87.1%), followed by Kato-Katz (84.4%), Mini-FLOTAC FS2 (61.3%) and McMaster (48.3%). The difference in helminth detection among the three techniques was statistically significant. Mini-FLOTAC FS7 and Kato-Katz method detected significantly more infections than McMaster (p < 0.05) for *A. lumbricoides* diagnosis. Mini-FLOTAC FS2 was more sensitive than both McMaster and Kato-Katz for *H.nana* (p < 0.05). Regarding the two flotation solutions, FS2 was more sensitive than FS7 for *H.nana* (92.7% vs 77.8%), but the latter was more sensitive for *A.lumbricoides* (87.1% vs 61.3%), although the differences were not statistically significant. The performance of the test on negative subjects (NPV) was high: > 85% for H. nana and > 90% for *A lumbricoides* with all methods. NPV for *H.nana* with Mini-FLOTAC was significantly higher than with Kato-Katz and McMaster method. However, NPV is greatly influenced by the prevalence of infection and its results may vary in other settings with different STH distribution. The prevalence of the other helminths was not high enough for statistical analyses.

**Table 1:** Sensitivity and negative predictive value (NPV) of each method, considering all methods combined as gold standard

**Method**	***H. nana***		***A. lumbricoides***	
	**N [Sensitivity (95% CI)]**	**NPV (95% CI)**	**N [Sensitivity (95% CI)]**	**NPV (95% CI)**
Kato-Katz	22/193 [49.0 (33.2-64.6)]	86.5 (81.1-92.0)	27/193 [84.4 (70.2-98.5) **]	96.7 (94.1-99.9)
McMaster	28/193 [60.5 (44.7-76.2)]	89.7 (84.8-94.6)	17/193 [48.3 (28.4-68.2)]	91.5 (87.1-95.9)
Mini-FLOTAC (FS2)	42/193 [92.7 (83.5-100.0)*]	98.0 (95.5-100.0)	20/193 [61.3 (42.5-80.1)]	93.1 (89.0-97.1)
Mini-FLOTAC (FS7)	35/193 [77.8 (64.5-91.0)]	93.7 (89.6-97.8)	28/193 [87.1 (73.7-100)**]	97.6 (94.9-100.0)

*Significant difference (p < 0.05) with McMaster and with Kato-Katz.

**Significant difference (p < 0.05) with McMaster.

The KI for agreement among techniques is shown in Table 2. KI was moderate for *H.nana* between Kato-Katz thick smear and Mini-FLOTAC FS7 and McMaster method (0.57 and 0.58 respectively); it was substantial between Kato-Katz and FS2 (0.63), McMaster and Mini-FLOTAC FS2 and FS7 (0.65 and 0.71, respectively) and between Mini-FLOTAC FS2 and FS7 (0.85). For *A.lumbricoides* infection the agreement was moderate between Kato-Katz and McMaster (0.59) and between McMaster and Mini-FLOTAC FS7 (0.57), was substantial between Kato-Katz and Mini-FLOTAC FS2 (0.78), McMaster and Mini-FLOTAC FS2 (0.67) and between Mini-FLOTAC FS2 and FS7 (0.76); it was almost perfect between Kato-Katz and Mini-FLOTAC FS7 (0.98).

**Table 2:** Kappa index for agreement among the diagnostic techniques

***H. nana***	**KK**	**McM**	**MFFS2**	**MFFS7**
**KK**		0.58	0.63	0.57
**McM**	0.58		0.65	0.71
**MFFS2**	0.63	0.65		0.85
**MFFS7**	0.57	0.71	0.85	
***A. lumbricoides***	**KK**	**McM**	**MFFS2**	**MFFS7**
**KK**		0.59	0.78	0.98
**McM**	0.59		0.67	0.57
**MFFS2**	0.78	0.67		0.76
**MFFS7**	0.98	0.57	0.76	

KK: Kato-Katz; McM: McMaster; MFFS2: Mini-FLOTAC with FS2 solution; MFFS7: Mini-FLOTAC with FS7 solution.

As the three techniques are quantitative diagnostic methods, comparison among EPG was also calculated and data are shown in Table 3. Mini-FLOTAC FS2 detected the highest number of EPG for *H.nana* (904), followed by Mini-FLOTAC FS2, McMaster and Kato-Katz (569, 457 and 111 EPG, respectively). For *A.lumbricoides* Kato-Katz detected the highest number of EPG (1315) followed by Mini-FLOTAC FS2 (1177 EPG), McMaster (995 EPG) and Mini-FLOTAC FS7 (643 EPG). Due to the large variance, the EPG values were not statistically different among the three methods.

**Table 3:** Eggs per gram (EPG) of faeces (arithmetic mean and standard deviation) for the three diagnostic techniques

	**Kato-Katz**	**McMaster**	**Mini-FLOTAC FS2**	**Mini-FLOTAC FS7**
	**EPG (SD)**	**EPG (SD)**	**EPG (SD)**	**EPG (SD)**
***H. nana***	111 (±600)	457(±3451)	904 (±15041)	569 (±4363)
***A. lumbricoides***	1315 (±7145)	995 (±6055)	1177 (±6928)	643 (±3323)

As shown in Additional file 1: Table S1, for *A.lumbricoides* light infections were the most frequent to be missed, in fact 12 negative samples at the McMaster resulted positive for light infections with the Kato-Katz, 7 negative with the Kato-Katz resulted positive for light infections with the Mini-FLOTAC FS2, and 6 negative with McMaster were positive for light infections with Mini-FLOTAC FS2; between Mini-FLOTAC FS2 and Mini-FLOTAC FS7, 9 negative with the former solution were positive for light infections with the latter.

### Technique feasibility

**Figure 2:**
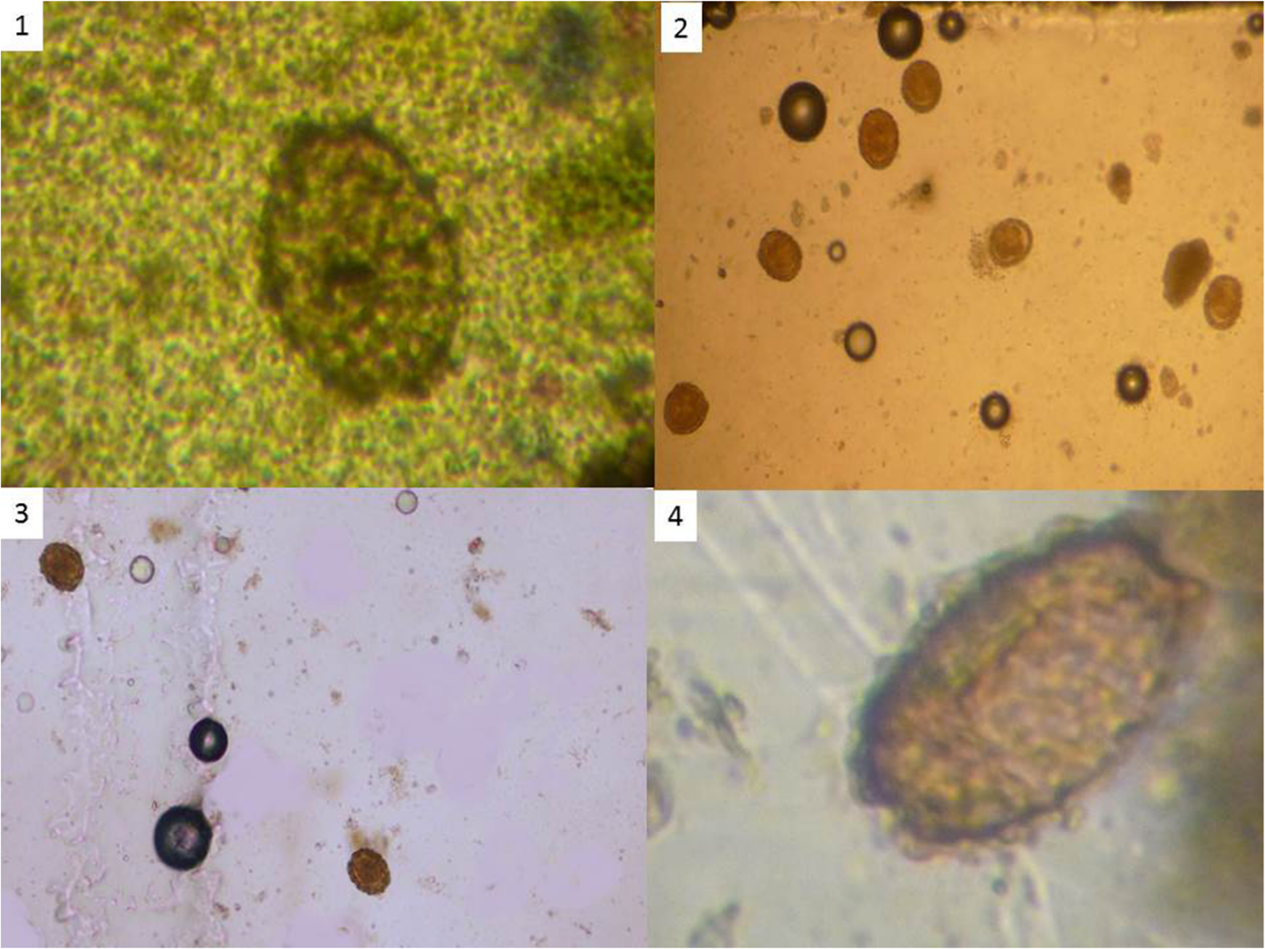
**Pictures of *Ascaris lumbricoides *** **with: 1) Kato-Katz thick smear, 2) McMaster method, 3) Mini-FLOTAC FS2 and 4) Mini-FLOTAC FS7.**

Overall, the most time-consuming method to be processed was Kato-Katz (mean time 48 min/sample), followed by the Mini-FLOTAC (13 min/sample). McMaster was the quickest to process (7 min/sample). Kato-Katz was the slowest to examine (10 min/sample), followed by the Mini-FLOTAC technique with FS7 (7 min/sample). The McMaster and Mini-FLOTAC with FS2 slides were the quickest to read (5 min/sample).The waiting time for the clarification of the eggs for Kato-Katz and for the floatation of the eggs for the Mini-FLOTAC were the factors that accounted most significantly for the length of processing single samples. It must be underlined, however, that for both techniques, when processing multiple, samples the time (min/slide) decreased significantly as the reading of the slide(s) was done while awaiting the cleansing of subsequent samples. Reading time was faster for the McMaster and Mini-FLOTAC FS2 as they were clearer slides, whilst Mini-FLOTAC FS7 and the Kato-Katz were slides more difficult to read due to the presence of debris and artefacts. Examples of slides with helminth eggs with the three different methods are illustrated in Figure 2. Prior to the study, the laboratory staff was not acquainted with the Kato-Katz or with the Mini-FLOTAC, and used a different protocol for the McMaster. All three techniques were easily taught (training took about one day each) and the staff performed all three methods with good quality after such short training.

## Discussion

The Mini-FLOTAC turned out to be an innovative, sensitive and low cost technique in the scenario of intestinal helminths diagnosis; it has been recently launched, so some aspects are still on trial, but recent studies revealed a potentially reliable diagnostic tool [15,16]. Mini-FLOTAC has been compared with the traditionally recommended quantitative techniques, both separately [16] and, in this work, together. As shown by these results, it may be considered a valid alternative to Kato-Katz, although in this study it was only possible to test for *A. lumbricoides* as the prevalence of the other two major STH, *T. trichiura* and hookworms, was too low for a meaningful evaluation. As for the comparison with McMaster method, Mini-FLOTAC proved to be more sensitive for all helminths, especially for *A. lumbricoides* and *H. nana*. This is the first study that reports the detection of *H. nana* with the Mini-FLOTAC and was shown to be a sensitive method for this particular parasite. Mini-FLOTAC also counted more eggs than Kato-Katz, which is not the best technique to assess intensity of infection of *H. nana*. One possible explanation could be *that H. nana* eggs are very small (30–40 μm) and yellowish/transparent and therefore could not easily be seen in the Kato-Katz. Rather they are well detected upon flotation in the Mini-FLOTAC. Even if *H. nana* is not included in helminth control programmes, studies conducted showed its pathogenicity and occasionally severe symptoms linked to this infection. It would be interesting, in the light of this reported data, to further investigate *H.nana* epidemiology, perhaps using the Mini-FLOTAC, which was demonstrated to be a sensitive diagnostic method for its detection [17-19]. The difference between the two floatation solutions even if present was not statistically significant, nor was the difference in egg counts calculated by the three methods. These data suggest that for public health surveys, Mini-FLOTAC with FS2 would be the optimal diagnostic tool, as it detects as many eggs as the other quantitative methods but it is more sensitive than both other techniques for *H. nana* and is more sensitive than the McMaster for *A. lumbricoides*. Advantages of the Mini-FLOTAC are its relatively quick processing and read out and its good sensitivity which compete with the McMaster (simpler but less sensitive) and with the Kato-Katz (as sensitive but more time-consuming).

Another added value of the Mini-FLOTAC is that it works in a “closed” system (using Fill-FLOTAC) and that faeces can be preserved with 5% formalin in order to guarantee the safety of the operator as well as the possibility of examining the samples up to one week after their collection. Regarding floatation solutions, FS7 is less easily available as zinc sulphate is not cheap and relatively difficult to find in low-resources settings, whilst FS2, which is based on saturated salt solution, is cheap and easily accessible. Regarding feasibility, when multiple faecal samples are examined in series, the processing time and reading differences of the three methods flatten, and are more or less similar among the three techniques, with a slight advantage for the McMaster. Among all determinants influencing the length of each technique, faecal egg count was the most relevant, especially for Kato-Katz.

In situations where helminth control strategies aim at control of transmission and eventually elimination of STH, the need of a diagnostic tool easy to use in the field and able to detect even very light infections is evident. The need of new sensitive diagnostic methods has been partially met by the introduction of the FLOTAC technique for direct diagnosis [10], and molecular diagnostic tool such as multiplex [20]. However, the advantage of the higher sensitivity of the FLOTAC technique is counterbalanced by its higher cost and longer processing. The field use of molecular techniques has been improved, but still requires technology, which is not yet routinely available in resource-limited settings [13,21].

The major limitation of this study is the low prevalence of helminth infections which hinders the full evaluation of the Mini-FLOTAC against a wide range of intestinal parasites. It would be useful, therefore, to plan other studies to compare these three techniques in areas where the prevalence of other STH is higher. A higher prevalence of STH in this area, especially hookworm was reported [4-6,13] a few years back, but since then an effective deworming project based on the distribution of albendazole and ivermectin based on a primary health care approach [22] has been carried out, and the prevalence, as shown in our data, has significantly decreased. Moreover, the assessment of Mini-FLOTAC performance on preserved samples over time would be useful, especially in settings where days/weeks might elapse between collection and processing of the samples.

## Conclusions

The ideal direct diagnostic method in helminthology should combine ease of use, affordability, and relatively good sensitivity, especially in quantitating egg counts. The data presented show that all three techniques tested in this study match these criteria, with a slight advantage for the Mini-FLOTAC with FS2. The techniques evaluated in this study should be recommended for STH mapping purposes, monitoring drug efficacy and impact of STH control programme interventions in endemic countries.

## Competing interests

Mini-FLOTAC and Fill-FLOTAC were invented and patented by Prof. Giuseppe Cringoli, University of Naples Federico II, Italy. However, there was no conflict of interest in the study.

## Authors’ contributions

BB, MA, RB, GC and AK designed the project. RC, LR made statistical analysis. BB, PC, EV, MJ carried out the work field and laboratory analyses. GC, RB financially supported the project. BB, MA, AL wrote the paper. All co-authors gave important contributions in revising the manuscript and answering the reviewers. All authors read and approved the final version of the manuscript.

## Supplementary Material

Additional file 1: Table S1 2×2 contingency table of A. lumbricoides intensity of infections with the three diagnostic methods. Click here for file
