# Supplementary material for: Mini-FLOTAC, Kato-Katz and McMaster: three methods, one goal; highlights from north Argentina
Source: Parasit Vectors. 2014 Jun 14;7:271. doi: 10.1186/1756-3305-7-271 (PMC4074144; doi:10.1186/1756-3305-7-271)

Additional file 1: Table S1. 2x2 contingency table of *A.lumbricoides* intensity of infections with the three diagnostic methods.


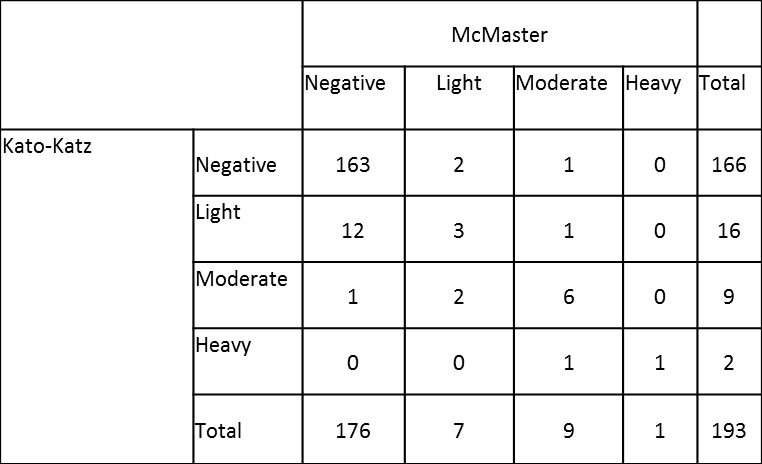

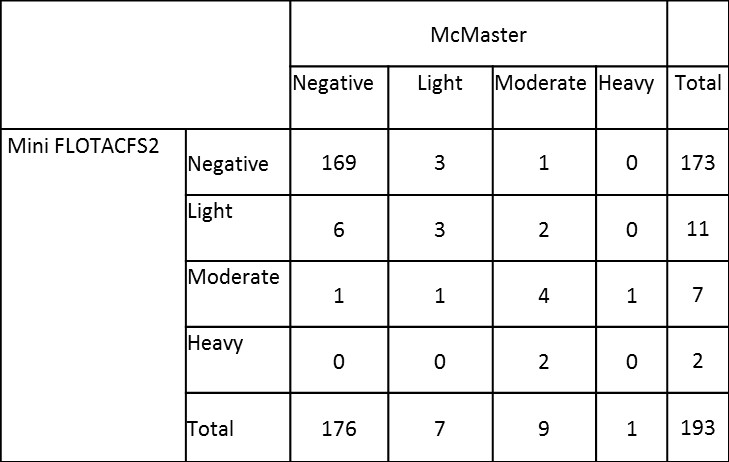


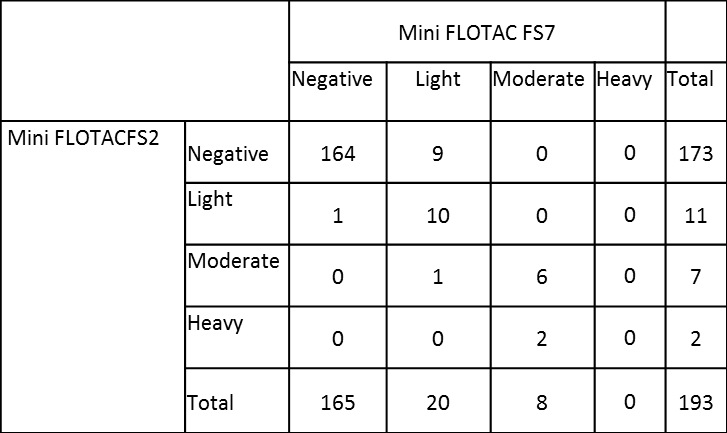

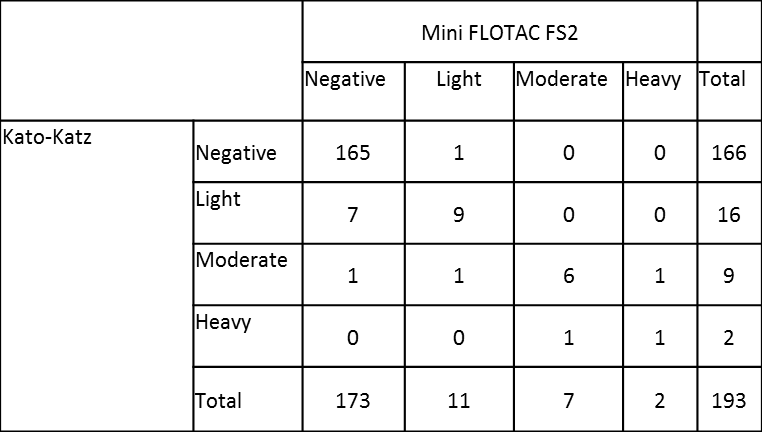

Supplement: Additional file 1: Table S1 — 2×2 contingency table of A. lumbricoides intensity of infections with the three diagnostic methods. [file 1756-3305-7-271-S1.doc]
